# Association of school neighbourhood socioeconomic disadvantage and teaching staff’s risk of violence at work

**DOI:** 10.1177/14034948241252232

**Published:** 2024-06-10

**Authors:** Jenni Ervasti, Jaana Pentti, Ville Aalto, Maarit Kauppi, Marianna Virtanen, Mika Kivimäki, Jussi Vahtera

**Affiliations:** 1 Finnish Institute of Occupational Health, Helsinki, Finland; 2 Clinicum, Faculty of Medicine, University of Helsinki, Finland; 3 Department of Public Health, University of Turku, Turku, Finland; 4 Centre for Population Health Research, University of Turku and Turku University Hospital, Turku, Finland; 5 School of Educational Sciences and Psychology, University of Eastern Finland, Joensuu, Finland; 6 Division of Insurance Medicine, Department of Clinical Neuroscience, Karolinska Institutet, Stockholm, Sweden; 7 Department of Mental Health of Older People, Faculty of Brain Sciences, University College London, London, UK

**Keywords:** area-level indicator, multi-level analysis, neighbourhood disadvantage, teaching staff, violence at work, workplace psychosocial resource

## Abstract

**Aim:**

The aim of this study was to determine the association between neighbourhood socioeconomic disadvantage and teaching staff’s risk of workplace violence and whether workplace psychosocial resources can act as effect modifiers.

**Methods:**

Primary school teaching staff in the six largest cities in Finland responded to a survey in 2018 and were linked to information on school neighbourhood disadvantage obtained from the national grid database (*n* = 3984).

**Results:**

After adjustment for confounders, staff working in schools located in the most disadvantaged neighbourhoods had a 1.2-fold (95% confidence interval 1.07–1.35) risk of encountering violence or threat of violence compared with staff working in the most advantaged neighbourhoods. The association was less marked in schools with strong support from colleagues (risk ratio 1.14, 95% confidence interval (95% CI) 0.98–1.32 for high support versus 1.23, 95% CI 1.07–1.43 for low/intermediate support), a strong culture of collaboration (1.08, 95% CI 0.93–1.26 versus 1.31, 95% CI 1.12–1.53), high leadership quality (1.12, 95% CI 0.96–1.31 versus 1.29, 95% CI 1.08–1.54), and high organizational justice (1.09, 95% CI 0.91–1.32 versus 1.29, 95% CI 1.09–1.52).

**Conclusions:**

**The association between school neighbourhood and teaching staff’s risk of violence was weaker in schools with high workplace psychosocial resources, suggesting that targeting these factors might help in minimizing violence at schools, but future intervention studies are needed to confirm or refute this hypothesis.**

## Background

The risk of violence and threat of violence at work is higher for teachers compared with the average working population [1,2]. In 2022 in Finland, 79% of special education teachers, and 60 % of classroom teachers reported having experienced a violent of threatening situation at work within previous 12 months [1]. A Canadian study from 2011 reported 80% lifetime prevalence of violence in teachers [2], whereas a meta-analysis combining 24 studies providing estimates of violence reported by teachers within previous 24 months found the prevalence range from 20% to 75% [3]. Workplace violence can include mental abuse, physical violence, such as hitting and kicking, throwing or breaking things and threatening with a weapon. Of the perpetrators of violence towards teachers, 90% are pupils [4].

Workplace violence can have several adverse consequences, such as sleep problems [5], mental health problems [6-8], poorer general health [9], poorer work ability [10] and even suicidality [11]. Employment in human service occupations is linked with higher risk of sickness absence [12]. This excess risk was explained by high emotional demands, low worktime control, and exposure to workplace violence [12]. Previous studies have found high job demands and low social support to be associated with increased risk of mental abuse and property offences reported by teachers [13], and school characteristics of high task variety, high creativity, high turnover rate and low decision latitude among teachers associated with higher odds of increased violence [14]. Studies also suggest that a history of violence may increase the risk of future violence [15], and that there is large between-school variance in the prevalence of physical violence [6]. These findings indicate that there may be school contextual factors that predispose to violence. In a case-control study on school contextual factors, the odds of violence against teachers were higher in smaller schools than in bigger schools, in schools with inadequate resources and building safety and in environments with physical barriers [16]. The odds were also increased in schools that had fewer routine locker searches and accessible exits [16].

Many of the identified risk characteristics may cluster into disadvantaged neighbourhoods through lower quality of local services, lower parental interest in children’s school performance, less favourable peer influence or greater exposure to crime and violence [17,18]. There are theories proposing that a child’s residential area affects their development. This can happen through sustained interaction between child’s characteristics and their neighbourhood or through poverty-driven inequalities. Social epidemiological theories suggest that socioeconomic status (SES), social networks and power divisions are the fundamental causes of ill-health [19]. In a pioneering study, informal social control, cohesion and trust within a neighbourhood were identified as robust predictors of lower rates of violence [20]. School neighbourhood disadvantage has been linked to higher risk of heavy alcohol consumption, poorer psychosocial work environment, higher risk of sickness absence and lower organizational (school) commitment among teachers [21-23]. However, we are not aware of previous studies examining whether school neighbourhood socioeconomic disadvantage was associated with teaching staff’s risk of violence or threat of violence.

We studied the association between school neighbourhood socioeconomic disadvantage and teaching staff’s reports of workplace violence, controlling for an array of municipality- and school-level confounders and individual teacher characteristics. Moreover, to identify potential modifiable factors for future interventions aimed at reducing the risk of violence at schools, we examined whether school-level favourable psychosocial work environment, i.e., culture of collaboration, strong support from colleagues, high leadership quality and organizational justice, may attenuate the association between school neighbourhood disadvantage and violence and threat of violence against teaching staff.

## Methods

### Study population

Study participants were from the Finnish Public Sector (FPS) study [21], and included teaching staff working in primary schools (grades 1–6) in the six largest cities in Finland, who responded to the FPS questionnaire survey in 2018 (teaching staff response rate 77%). Survey responses were linked to grid-based data on socioeconomic characteristics of each school neighbourhood provided by Statistics Finland. Participants with missing information on main exposure or outcome variables were omitted (*n* = 50), resulting in an analytic sample of 3984 participants in 193 schools. The FPS study was approved by the ethics committee of Helsinki and Uusimaa Hospital District (HUS/1210/2016).

### Outcome: violence at work

Violence at work was measured with the following question: ‘Have any of the following violent or threatening confrontations involving clients happened to you over the past 12 months? (1) Throwing or breaking things; (2) Mental abuse (e.g., verbal threats); (3) Physical violence (e.g., hitting, kicking); (4) Threatening with a weapon (firearm, edged weapon, striking weapon)?’ ‘Yes’ to any kind of violence was coded as 1, and ‘No’ to all was coded as 0. If the respondent replied ‘Yes’, we asked how often confrontations had happened (daily, weekly, monthly, less frequently). The frequency of violence was coded as 0 = no violence or threat of violence; 1 = less frequently; 2 = monthly; 3 = weekly/daily [5,6,14,24]. In sensitivity analysis, we also examined the types of violence separately.

### Exposure: school neighbourhood socioeconomic disadvantage

We obtained school addresses from the National Register of Public Corporations. The characterization of the school neighbourhood was based on a grid database containing Statistics Finland’s coordinate-based data in 2019 calculated by map grid. It covers data describing the structure of the population, including information on education, main type of activity (employed, unemployed), household income within 250 × 250 m^2^ grids. A school neighbourhood from which the students come to school was defined as 1250 × 1250 m^2^ areas, with the school in the innermost grid in this study. The schools were linked to neighbourhoods by geocoded school addresses.

As in previous studies [25,26], we calculated a standardized index for the socioeconomic environments of each school neighbourhood using the grid database information on the average annual income of households (coded as additive inverse), education attainment (percentage of people aged > 18 years with low education), and unemployment rate (unemployed persons of the economically active population). Higher scores on the continuous index denote greater deprivation. For each of the three variables, we derived a standardized *z*-score (national mean = 0, standard deviation = 1). School neighbourhood socioeconomic disadvantage scores were then calculated by taking the mean value across all *z*-scores when the *z*-score for at least one of the indicators was available and categorized the mean area disadvantage to four levels using −0.5, 0 and +0.5 as cut-off points. In addition to a categorized variable, we used a continuous score of school neighbourhood socioeconomic disadvantage.

### Covariates

We treated municipality (i.e., six cities coded as six-class categorical variable), school neighbourhood population density and school size (as indexed by number of teachers) as area-level confounders. Individual-level confounders from the employer register data were sex (man/woman), age (continuous), job contract (permanent/temporary) and occupation (principal, other managerial position/lecturer, subject teacher/classroom teacher, special education teacher/classroom assistant).

### Effect modifiers

We studied whether workplace psychosocial resources acted as effect modifiers modifying the association between school neighbourhood socioeconomic disadvantage and teaching staff’s reports of violence at work. The chosen workplace psychosocial resources were derived from previous research and described in detail elsewhere [27]. In short, psychosocial resources consist of support from colleagues (one item), culture of collaboration (two items), leadership quality (four items) and procedural organizational justice (four items). The response for these items was provided on a scale ranging from 1 to 5, where 1 = completely disagree/very little, that is, lowest amount of a resource, 2 = somewhat disagree/fairly little, 3 = not agree nor disagree/some, 4 = somewhat agree/quite a lot, 5 = totally agree/very much indicating the highest amount of a resource. These measures were derived from teaching staff’s survey responses and aggregated to school level.

As there are no established or theory-based cut-offs for the categorization of the psychosocial resource measures, we used a distribution-based categorization, dichotomizing the variables into ‘low to intermediate’ versus ‘high’ at median: support from colleagues, Q1 = 3.9, median (Q2) = 4.10, Q3 = 4.4; culture of cooperation, Q1 = 3.5 median (Q2) = 3.71, Q3 = 4.0; leadership quality, Q1 = 3.6, median (Q2) = 3.95, Q3 = 4.2; procedural justice, Q1 = 3.2, median (Q2) = 3.49, Q3 = 3.7. As the median value for all these measures exceeded the intermediate response option (i.e., 3), we labelled the dichotomized categories as ‘low to intermediate’ versus ‘high’. This median split provided equal-sized groups for comparisons and optimized statistical power.

### Statistical analysis

We used frequencies with chi-squared tests for nominal scale data and means with *t*-tests for continuous and ordinal scale data to describe the study population and differences between teaching staff reporting violence and those who did not. Due to the hierarchical nature of the data (staff nested in schools) we analysed the data using Poisson regression analysis, with school as repeated subject within the generalized estimating equations method. This method takes into account the correlation between violence reports within schools. Risk ratios and risk differences with their 95% confidence intervals were first adjusted for municipality, neighbourhood population density and school size. The most adjusted model also included participants’ age, sex, job contract and occupation. As a sensitivity analysis, we examined whether excluding classroom/teaching assistants, who lack pedagogical training and could thus have a higher likelihood of violence, affected the result.

Second, we examined the frequency of violence. Here, we stratified the analysis by violence frequency, each time using ‘no violence’ as the reference group.

Third, we examined the possible effect modification of workplace psychosocial resources by testing psychosocial resources × neighbourhood socioeconomic-disadvantage interaction and calculating contrast estimates for the dichotomized level of support from colleagues, culture of cooperation, quality of leadership and procedural justice. As a sensitivity analysis, we created a sum score of workplace psychosocial resources based on the dichotomized resources (0 = all resources low; 1–2 resources high; 3–4 resources high).

All analyses were done using SAS version 14.3.

## Results

A total of 61% of the teaching staff reported any kind of violence or threat of violence during the previous 12 months. When stratified by type of violence, 50% reported incidents involving breaking or throwing things, 41 % reported mental abuse, 31% reported physical violence and 2% reported incidents involving a weapon. Reports of violence were more common in neighbourhoods with higher population density, and in larger schools. There was a marginal difference in means of workplace psychosocial resources so that they were marginally better in teachers not reporting violence. Women, younger teachers, those with temporary job contracts, special education teachers and classroom/teaching assistants were over-represented among those reporting violence (Table I).

**Table I.** Characteristics of teaching staff by reporting violence or threat of violence at work. Numbers are *N* of participants (percentage) unless otherwise stated.

	Violence or threat of violence		*p* for difference between groups
	No *N* = 1561 (39%)	Yes *N* = 2423 (61%)	
School neighbourhood (1250 × 1250 m^2^) characteristics			
Population density, mean (SD)	5632 (4242)	5861 (4088)	0.09
School characteristics			
School size, mean *N* of teachers (SD)	33.9 (20.3)	38.0 (25.1)	<0.001
Support from colleagues at school, mean (SD)	4.11 (0.3)	4.07 (0.4)	0.002
Culture of collaboration at school, mean (SD)	3.78 (0.3)	3.75 (0.3)	0.003
Leadership quality at school, mean (SD)	3.91 (0.4)	3.87 (0.5)	0.009
Procedural justice at school, mean (SD)	3.49 (0.4)	3.44 (0.4)	<0.001
Teaching staff characteristics			
Men	325 (21)	369 (15)	
Women	1236 (79)	2054 (85)	<0.001
Age, mean (SD)	46.5 (9.6)	44.7 (9.9)	<0.001
Permanent job contract	1306 (84)	1886 (78)	
Temporary job contract	255 (16)	537 (22)	<0.001
Principal/other managerial position	65 (4)	82 (3)	
Lecturer, subject teacher	345 (22)	489 (20)	
Classroom teacher	959 (61)	1121 (46)	
Special education teacher	118 (8)	439 (18)	
Classroom/teaching assistant	74 (5)	292 (12)	<0.001

SD, standard deviation.

In the most disadvantaged neighbourhoods, 74 % of the teaching staff reported violence, while in the most affluent neighbourhoods the corresponding figure was 56 %. After adjusting for area, school and individual-level covariates, teaching staff in schools located in the most disadvantaged neighbourhoods had a 1.2-fold (95% confidence interval (95% CI) 1.07–1.35) risk of encountering violence or threat of violence than staff in schools located in the most advantaged neighbourhoods. The corresponding/adjusted risk difference for encountering violence at school was 15% for teaching staff working in schools in the most disadvantaged neighbourhoods compared with staff working in schools in the most advantaged neighbourhoods (Table II). The association remained when excluding classroom/teaching assistants (model 3: risk ratio (RR) = 1.24, 95% CI 1.10–1.41, data not shown in tables). We performed a sensitivity analysis by type of violence presented in Supplementary Table I. The results were largely similar to main results using all types of violence combined.

**Table II.** The association between school neighbourhood socioeconomic disadvantage (socioeconomic status, SES) and workplace violence or threat of violence among teaching staff (*N* = 3984). Multi-level modelling with school as repeated subject (secon-level variable/random effect).

	Violent encounter in past 12 months *N* (%)	Model 1^*^				Model 2^**^				Model 3^***^			
		RR	95% CI	RD (%)	95% CI	RR	95% CI	RD (%)	95% CI	RR	95% CI	RD (%)	95% CI
Highest SES	633 (56)	1		0		1		0		1		0	
(2)	956 (60)	1.07	0.94–1.21	3.7	–3.6–11.1	1.07	0.94–1.21	3.6	–3.9–11.1	1.05	0.93–1.18	3.7	–3.5–10.9
(3)	588 (62)	1.10	0.98–1.24	5.5	–1.5–12.6	1.12	1.00–1.25	6.1	–0.04–12.5	1.08	0.97–1.20	4.7	–1.6–11.2
Lowest SES	246 (74)	**1.25**	**1.08–1.45**	**14.0**	**4.3–23.7**	**1.28**	**1.13–1.44**	**15.4**	**7.9–22.9**	**1.20**	**1.07–1.35**	**15.1**	**7.6–22.7**
Continuous SES^†^		**1.15**	**1.05–1.25**			**1.19**	**1.10–1.29**			**1.13**	**1.05–1.22**		

* Statistically significant associations are bolded. Model 1 is unadjusted

** Model 2 is adjusted for municipality, neighbourhood population density, and school size

*** Model 3 is adjusted as Model 2 and for teacher characteristics

† School neighbourhood SES per 1 standard deviation increase in the neighbourhood disadvantage score.

RR: risk ratio (relative risk); CI: confidence interval; RD: risk difference (absolute risk).

The association varied by the frequency of violence. As shown in Table III, the risk of less than monthly (RR = 1.24, 95% CI 1.02–1.29) and monthly (RR = 1.64, 95% CI 1.29–2.08) violence was higher for teaching staff in schools located in the most disadvantaged neighbourhoods when compared with those in the most advantaged neighbourhoods. However, the association diluted for weekly/daily violence.

**Table III.** The association between school neighbourhood socioeconomic disadvantage (socioeconomic status, SES) and workplace violence or threat of violence among teaching staff (*N* = 3984) by violence frequency (reference = no violence).

Violence frequency		Less than monthly		Monthly		Weekly/daily	
		RR	95% CI	RR	95% CI	RR	95% CI
Model 3^*^	Highest SES	1		1		1	
	2.	1.08	0.91–1.27	1.17	0.92–1.50	1.05	0.81–1.35
	3.	1.10	0.94–1.29	1.18	0.92–1.51	1.12	0.88–1.41
	Lowest SES	**1.24**	**1.02–1.51**	**1.64**	**1.29–2.08**	1.04	0.72–1.49
	Continuous SES^†^	**1.15**	**1.02–1.29**	**1.39**	**1.19–1.64**	1.15	0.94–1.40

*** Statistically significant associations are bolded. Model 3 is adjusted for municipality, neighbourhood population density, school size and for teacher characteristics

† School neighbourhood SES per 1 standard deviation increase in the neighbourhood disadvantage score.

RR: risk ratio (relative risk); CI: confidence interval.

**Figure 1.**
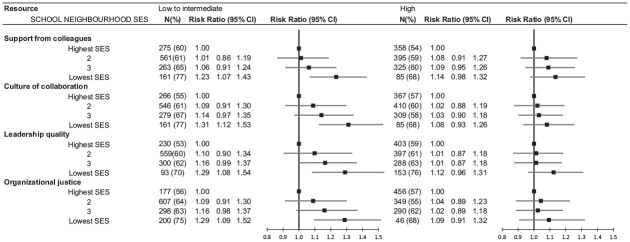
The association between school neighbourhood socioeconomic disadvantage (socioeconomic status, SES) and workplace violence or threat of violence among teachers in analysis stratified by workplace psychosocial resources. CI: confidence interval; RR: risk ratio; SES: socioeconomic status.

In the analysis stratified by workplace psychosocial resources, the relative risk associated with the most disadvantaged neighbourhoods diluted in schools with above median level of support from colleagues, culture of collaboration, leadership quality and organizational justice. The associations remained in schools with lower than median level of workplace psychosocial resources, with relative risks ranging from 1.23 to 1.31. (Figure 1, Supplementary Table II) Despite the differences found in the stratified analyses, all interaction terms were non-significant (*p* > 0.20).

In supplementary analysis, we also show the results using the sum score of workplace psychosocial resources. We found that for 44% of the teaching staff, 3–4 of the workplace psychosocial resources were high, and the association between school neighbourhood disadvantage and teaching staff’s risk of workplace violence diluted. The association remained in schools in the most disadvantaged neighbourhoods when all workplace psychosocial resources were low (RR = 1.28, 95% CI 1.02–1.61) and when only one or two of the resources were high (RR = 1.19, 95% CI 1.01–1.41). An association emerged when comparing the second most disadvantaged school neighbourhoods with the most advantageous neighbourhoods when all resources were low to intermediate (RR = 1.40, 95% CI 1.08–1.81) (Supplementary Table III). The interaction term school neighbourhood SES × psychosocial resource sum score was *p* = 0.44.

## Discussion

We found that school neighbourhood socioeconomic disadvantage was associated with teaching staff’s risk of violence or threat of violence encountered at school. However, in schools characterized by high coworker support, culture of collaboration, leadership quality and procedural justice, the association was diluted, suggesting that these workplace psychosocial resources might buffer against violence at school.

We are not aware of previous studies on the association between school neighbourhood disadvantage and teaching staff’s risk of encountering workplace violence. Our results are in line with previous research that found school contextual factors, including inadequate resources, to be associated with higher odds of violence against teachers [16]. Our finding that workplace psychosocial resources may moderate the association between neighbourhood disadvantage and violence risk is consistent with previous studies, which reported associations between lack of psychosocial resources and risk of violence [13,14,24]. These studies, however, did not consider the role of school neighbourhood disadvantage.

In our study, as many as 61% of the teaching staff reported having experienced violence or threat of violence during the past 12 months, but the risk varied between schools. Employers in Finland are mandated to ensure the safety and health of the employees. Workplace violence has been linked with several adverse health outcomes [7,8,11,28,29], emphasizing the need to identify modifiable factors that can help prevent violence at schools and in other workplaces. Previous research has linked job stress and other psychosocial risk factors with increased probability of workplace violence [24,30-32]. The relationship is likely to be bidirectional such that job stress leads to staff neglecting the early signs of aggression or being distracted, but also that violent encounters increase job stress and may adversely affect psychosocial work environment. Our findings showed that a favourable psychosocial work environment was associated with diluted excess risk of violence in disadvantaged school neighbourhoods. In schools with good leadership and management practices, the procedures and instructions on how to handle violent situations and how the aftermath and follow up in case of such incidents is handled, are likely to be better than in schools with poorer leadership and management.

In terms of why neighbourhood influences children’s violent behaviour, previous research suggests a pathway through social interactive mechanisms, such as safety and the likelihood that the community would intervene on children. While the most proximal environment, namely the family, seems most important, people, resources and opportunities within the residential area are also contributing to a child’s development [19].

The major strengths of the study were the large dataset from which the primary school teaching staff of the six largest cities were derived, and the linked, objective, grid-based data on school neighbourhood characteristics.

The limitations include the observational study design limiting the possibility for causal conclusions. It is possible that the increased risk of violence towards teaching staff in poorer neighbourhoods is at least partly explained by teaching staff selection, if for example, inexperienced teachers are selected to more disadvantaged school neighbourhoods. The model of positive discriminations in some Finnish cities increases, particularly classroom/teaching assistants in schools in poorer neighbourhoods, which might bias findings. However, we controlled for several individual characteristics, including age and job contract, and our results were robust to excluding classroom/teaching assistants, which decrease the possibility of bias in our results.

The response rate was 77%, which is considered high. However, 23% of the eligible population did not participate in our study. Given that the response rate varied between schools, it is possible that teachers in schools located in disadvantaged neighbourhoods were less inclined to respond to the survey. This could potentially have a diluting effect on our results. Moreover, the data on violence relied on self-reports, which can introduce several sources of bias. These include subjectivity in perceptions of what constitutes violence and potential recall bias, where a person may not accurately remember past events. In contrast, the measure of neighbourhood disadvantage was derived from recorded grid data, and the measures of workplace psychosocial resources were based on aggregated school-level data. These types of data help to mitigate common method bias, as they are not based on the individual participants’ perceptions.

The study was conducted in Finland, a Nordic welfare state with small neighbourhood socioeconomic differences. This may hamper the generalizability, but it can be assumed that even larger differences may be found in societies with larger socioeconomic disparity.

In conclusion, teaching staff who worked in schools located in disadvantaged neighbourhoods experienced more violence and threat of violence than those working in schools in more affluent areas. This association was weaker in schools with high workplace psychosocial resources, suggesting that targeting these factors might help in minimizing violence at schools. Future intervention studies are needed to confirm or refute this hypothesis.

## Supplemental Material

sj-docx-1-sjp-10.1177_14034948241252232 – Supplemental material for Association of school neighbourhood socioeconomic disadvantage and teaching staff’s risk of violence at work Supplemental material, sj-docx-1-sjp-10.1177_14034948241252232 for Association of school neighbourhood socioeconomic disadvantage and teaching staff’s risk of violence at work by Jenni Ervasti, Jaana Pentti, Ville Aalto, Maarit Kauppi, Marianna Virtanen, Mika Kivimäki and Jussi Vahtera in Scandinavian Journal of Public Health
