# Supplementary material for: Association of school neighbourhood socioeconomic disadvantage and teaching staff’s risk of violence at work
Source: Scand J Public Health. 2024 Jun 10;53(4):429–36. doi: 10.1177/14034948241252232 (PMC12048727; doi:10.1177/14034948241252232)
Supplement: sj-docx-1-sjp-10.1177_14034948241252232 – Supplemental material for Association of school neighbourhood socioeconomic disadvantage and teaching staff’s risk of violence at work [file sj-docx-1-sjp-10.1177_14034948241252232.docx]

**SUPPLEMENTARY MATERIAL**

**Supplementary Table 1.** The association between school neighbourhood socioeconomic disadvantage (socioeconomic status, SES) and types of violence at school reported by teaching staff (N=3984). RR=risk ratio (relative risk); CI=confidence interval; RD=risk difference (absolute risk). Multi-level modelling with school as repeated subject (2^nd^ level variable/random effect). Models are adjusted for municipality, neighbourhood population density, school size, and teacher characteristics (sex, age, job contract, teacher type).

|  | N (%) | Throwing or breaking things | | | | N (%) | Mental violence | | | | N (%) | Physical violence* | | N (%) | Weapon threat* | |
| --- | --- | --- | --- | --- | --- | --- | --- | --- | --- | --- | --- | --- | --- | --- | --- | --- |
|  |  | RR | 95% CI | RD (%) | 95% CI |  | RR | 95% CI | RD (%) | 95% CI |  | RR | 95% CI |  | RR | 95% CI |
| Highest SES | 518 (46) | 1 |  | 0 |  | 412 (37) | 1 |  | 0 |  | 294 (26) | 1 |  | 19 (2) | 1 |  |
| 2. | 762 (48) | 1.00 | 0.86-1.15 | 1.1 | -5.5, 7.9 | 637 (40) | 1.08 | 0.90-1.28 | 3.5 | -2.7, 9.7 | 494 (31) | 1.11 | 0.89-1.37 | 30 (2) | 0.98 | 0.50-1.91 |
| 3. | 497 (53) | 1.08 | 0.94-1.24 | 5.1 | -1.9, 12.2 | 377 (40) | 1.09 | 0.91-1.31 | 2.6 | -3.8, 8.9 | 321 (34) | 1.22 | 0.99-1.50 | 15 (2) | 0.76 | 0.39-1.50 |
| Lowest SES | 207 (62) | **1.20** | **1.04-1.38** | **13.6** | **5.5, 21.8** | 192 (58) | **1.41** | **1.20-1.67** | **15.8** | **8.1, 23.7** | 138 (42) | 1.28 | 0.99-1.65 | 19 (6) | **2.41** | **1.05-5.54** |
| Continuous SES** |  | **1.14** | **1.04-1.26** |  |  |  | **1.23** | **1.09-1.39** |  |  |  | **1.26** | **1.08-1.46** |  | 1.47 | 0.80-2.72 |

* Risk difference model did not converge

** School neighbourhood socioeconomic status per 1 standard deviation increase in the neighbourhood disadvantage score

**Supplementary Table 2.** The association between school neighbourhood socioeconomic disadvantage (continuous variable) and workplace violence or threat of violence among teachers in analysis stratified by workplace psychosocial resources. RR=risk ratio; CI=confidence interval

|  |  | **Support from colleagues** | | | | **Culture of collaboration** | | | | **Leadership quality** | | | | **Organizational justice** | | | |
| --- | --- | --- | --- | --- | --- | --- | --- | --- | --- | --- | --- | --- | --- | --- | --- | --- | --- |
|  |  | Low/ intermediate | | High | | Low/ intermediate | | High | | Low/ intermediate | | High | | Low/ intermediate | | High | |
|  |  | N teachers | N schools | N teachers | N schools | N teachers | N schools | N teachers | N schools | N teachers | N schools | N teachers | N schools | N teachers | N schools | N teachers | N schools |
|  |  | 1990 | 78 | 1994 | 115 | 2000 | 79 | 1984 | 114 | 1982 | 82 | 2002 | 111 | 2005 | 81 | 1979 | 112 |
| Prevalence of violence | | 63% |  | 58% |  | 63% |  | 59% |  | 60% |  | 62% |  | 64% |  | 58% |  |
|  |  | RR | 95% CI | RR | 95% CI | RR | 95% CI | RR | 95% CI | RR | 95% CI | RR | 95% CI | RR | 95% CI | RR | 95% CI |
| Model 3* | Continuous SES† | **1.14** | **1.03-1.25** | **1.13** | **1.02-1.26** | **1.18** | **1.07-1.31** | 1.09 | 0.97-1.21 | **1.19** | **1.06-1.34** | 1.09 | 0.98-1.20 | **1.20** | **1.09-1.31** | 1.08 | 0.95-1.23 |

*** Model 3 is adjusted for municipality, neighbourhood population density, school size, and for teacher characteristics
† School neighbourhood socioeconomic status per 1 standard deviation increase in the neighbourhood disadvantage score

**Supplementary Table 3.** The association between school neighbourhood socioeconomic disadvantage (socioeconomic status, SES) and workplace violence or threat of violence among teachers by sum of workplace psychosocial resources. RR=risk ratio; CI=confidence interval

|  |  | **No resource high (28%)** | | **1-2 resources high (28%)** | | **3-4 resources high (44%)** | |
| --- | --- | --- | --- | --- | --- | --- | --- |
|  |  | N teachers | N schools | N teachers | N schools | N teachers | N schools |
|  |  | 1126 | 38 | 1100 | 52 | 1758 | 103 |
|  |  | **RR** | **95% CI** | **RR** | **95% CI** | **RR** | **95% CI** |
| Model 3* | Highest SES | 1 |  | 1 |  | 1 |  |
|  | 2. | 1.25 | 0.96-1.62 | 0.97 | 0.79-1.17 | 1.00 | 0.82-1.20 |
|  | 3. | **1.40** | **1.08-1.81** | 0.95 | 0.76-1.19 | 1.00 | 0.86-1.17 |
|  | Lowest SES | **1.28** | **1.02-1.61** | **1.19** | **1.01-1.41** | 0.95 | 0.80-1.14 |
|  | Continuous† | **1.26** | **1.08-1.48** | 1.07 | 0.96-1.20 | 1.04 | 0.91-1.18 |

*** Model 3 is adjusted for municipality, neighbourhood population density, school size, and for teacher characteristics
† School neighbourhood socioeconomic status per 1 standard deviation increase in disadvantage score
